# Cytotoxic Capacity of SIV-Specific CD8^+^ T Cells against Primary Autologous Targets Correlates with Immune Control in SIV-Infected Rhesus Macaques

**DOI:** 10.1371/journal.ppat.1003195

**Published:** 2013-02-28

**Authors:** Daniel Mendoza, Stephen A. Migueles, Julia E. Rood, Bennett Peterson, Sarah Johnson, Nicole Doria-Rose, Douglas Schneider, Eva Rakasz, Matthew T. Trivett, Charles M. Trubey, Vicky Coalter, Claire W. Hallahan, David Watkins, Genoveffa Franchini, Jeffrey D. Lifson, Mark Connors

**Affiliations:** 1 HIV-Specific Immunity Section, Laboratory of Immunoregulation, National Institute of Allergy and Infectious Diseases, National Institutes of Health, Bethesda, Maryland, United States of America; 2 AIDS and Cancer Virus Program, SAIC-Frederick, Inc., Frederick National Laboratory for Cancer Research, Frederick, Maryland, United States of America; 3 Department of Pathology and Laboratory Medicine, University of Wisconsin-Madison, Madison, Wisconsin, United States of America; 4 Biostatistics Research Branch, National Institute of Allergy and Infectious Diseases, National Institutes of Health, Bethesda, Maryland, United States of America; 5 University of Miami Miller School of Medicine, Department of Pathology Clinical Research Building, Miami, Florida, United States of America; 6 Vaccine Branch, National Cancer Institute, National Institutes of Health, Bethesda, Maryland, United States of America; Emory University, United States of America

## Abstract

Although the study of non-human primates has resulted in important advances for understanding HIV-specific immunity, a clear correlate of immune control over simian immunodeficiency virus (SIV) replication has not been found to date. In this study, CD8^+^ T-cell cytotoxic capacity was examined to determine whether this function is a correlate of immune control in the rhesus macaque (RM) SIV infection model as has been suggested in chronic HIV infection. SIVmac251-infected human reverse transcriptase (hTERT)-transduced CD4^+^ T-cell clone targets were co-incubated with autologous macaque effector cells to measure infected CD4^+^ T-cell elimination (ICE). Twenty-three SIV-infected rhesus macaques with widely varying plasma viral RNA levels were evaluated in a blinded fashion. Nineteen of 23 subjects (83%) were correctly classified as long-term nonprogressor/elite controller (LTNP/EC), slow progressor, progressor or SIV-negative rhesus macaques based on measurements of ICE (weighted Kappa 0.75). LTNP/EC had higher median ICE than progressors (67.3% [22.0–91.7%] vs. 23.7% [0.0–58.0%], p = 0.002). In addition, significant correlations between ICE and viral load (r = −0.57, p = 0.01), and between granzyme B delivery and ICE (r = 0.89, p<0.001) were observed. Furthermore, the CD8^+^ T cells of LTNP/EC exhibited higher per-cell cytotoxic capacity than those of progressors (p = 0.004). These findings support that greater lytic granule loading of virus-specific CD8^+^ T cells and efficient delivery of active granzyme B to SIV-infected targets are associated with superior control of SIV infection in rhesus macaques, consistent with observations of HIV infection in humans. Therefore, such measurements appear to represent a correlate of control of viral replication in chronic SIV infection and their role as predictors of immunologic control in the vaccine setting should be evaluated.

## Author Summary

Clues regarding the features of effective immunity against lentiviruses have come from the study of non-human primates. We evaluated rhesus macaques infected with Simian Immunodeficiency Virus (SIV), a lentivirus closely related to Human Immunodeficiency Virus (HIV). In contrast to most SIV-infected rhesus macaques that develop progressive disease, a small proportion are able to control SIV replication and remain healthy for prolonged durations. In this study, we found that these long-term nonprogressor/elite controller (LTNP/EC) macaques have CD8^+^ T cells that are extremely effective at killing SIV-infected cells. It seems that this control is mediated by the efficient delivery of active granzyme B, a key molecule involved in the elimination of virus-infected cells. Furthermore, we correctly predicted the presence or absence of control of SIV infection in the majority of animals through measurements of the killing capacity of their CD8^+^ T cells. These findings indicate that measuring these functions could be used in the evaluation of vaccines against SIV in non-human primates.

## Introduction

Clues regarding the features of an effective cellular immune response capable of controlling a chronic lentiviral infection have come from humans who naturally restrict HIV replication referred to as long-term nonprogressors/elite controllers (LTNP/EC) [1]–[4]. LTNP/EC show an enrichment of some MHC class I alleles, particularly B*57 and B*27 [5]–[8], and their CD8^+^ T cell responses are focused on epitopes restricted by these alleles [6], [9]. HIV-specific CD8^+^ T cells of LTNP/EC display greater capacity to proliferate, upregulate granzyme (Gr) B and perforin expression, and suppress HIV replication or kill autologous HIV-infected CD4^+^ T cells *in vitro* compared to those of progressors [8], [10]–[13]. Our group has observed that delivery of active GrB to target cells resulting in efficient infected CD4^+^ T-cell elimination (ICE) clearly distinguishes LTNP/EC from untreated or treated progressors [12]–[14], which supports these measurements are clear correlates of immune control in HIV infection.

A subset of SIV-infected rhesus macaques behave as LTNP/EC manifesting similar features of effective immune system-mediated control of lentiviral infection. MHC class I alleles are associated with control of SIV infection, particularly Mamu B*08 and B*17 [15], [16]. The CD8^+^ T cells of LTNP/EC carrying these alleles preferentially recognize Mamu B08 and B17-bound SIV epitopes [17]. Furthermore, the 2–4 log increase in SIV plasma viremia seen after *in vivo* CD8^+^ T cell depletion in both LTNP/EC and progressors *in vivo* [18]–[20] provides further support that SIV is controlled by the CD8^+^ T cell response in these animals.

Despite these advances, there are no clear, generally agreed upon *in vitro* correlates of immune control of SIV infection and the precise mechanisms that underlie differences between immunologic control and lack of control over lentiviral infections remain incompletely understood. Some characteristics of the SIV-specific cellular immune responses have been reported as not correlating with immunologic control, including the magnitude or breadth of the CD8^+^ T cell response, epitope affinity or avidity, CD8^+^ T cell multi-functionality or cytokine secretion, CD8^+^ T cell phenotype, expression of PD-1 in CD8^+^ T cells, SIV epitopes recognized, or recognition of escape variant peptides [21]–[23]. Therefore, given our prior observation of correlations between *in vitro* GrB delivery or infected CD4^+^ T-cell elimination and *in vivo* control of HIV replication, it was of interest to measure CD8^+^ T-cell cytotoxic capacity, using similar assays, in the rhesus macaque model where there are clear examples of T-cell-mediated immune control over SIV in the setting of chronic infection or vaccination [24].

In the present study, we explored the cytotoxicity of SIV-specific CD8^+^ T cells against autologous SIV-infected CD4^+^ T-cell lines in rhesus macaques with progressive and nonprogressive SIV infection in a blinded fashion in an effort to identify a correlate of immune control in this model of lentiviral infection.

## Results

### Delivery of granzyme B to SIV-infected targets and infected CD4^+^ T-cell elimination (ICE) are detectable and can be used to predict the disease status of SIV-infected rhesus macaques

In humans, HIV-specific CD8^+^ T-cell cytotoxic capacity measured by GrB target cell activity and ICE are readily detectable in LTNP/EC, progressors and vaccinees [12]–[14]. One obstacle to adapting these assays to the rhesus macaque model is the requirement for 100–150 million PBMC, cell numbers that are not readily available from a single sampling of a rhesus macaque. Therefore, CD4^+^ T-cell lines immortalized by transduction with human telomerase reverse transcriptase (hTERT) were used to overcome limitations in the numbers of macaque cells needed as targets [25]. These cell lines can be expanded and maintained for prolonged periods *in vitro* with IL-2 and bi-weekly anti-CD3 monoclonal antibody stimulation with irradiated human PBMC and Epstein Barr virus-transformed B-cells as feeders [25]. Furthermore, the cell lines express surface markers similar to those of non-immortalized CD4^+^ T cells and can be infected with SIV [26].

**Figure 1:**
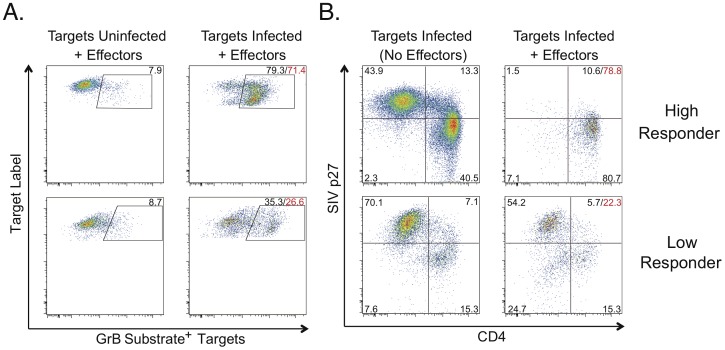
SIV-specific CD8^+^ T cell cytotoxicity measured by granzyme B delivery or Infected CD4 Elimination (ICE). **A.** The top panels show granzyme B (GrB) target cell activity representative of a “high responder”. The bottom panels show GrB target cell activity representative of a “low responder”. Values indicate percentages of targets with increased fluorescence due to GrB substrate cleavage. Background GrB target cell activity measured in response to uninfected targets (left column) was subtracted from responses measured against infected targets (right column) to determine net GrB target cell activity (red values). **B.** ICE values calculated based on p27 expression (sum of the upper quadrants) as described in the Methods, are shown in red for the same “high responder” (78.8%, top row) and “low responder” (22.3%, bottom row) as shown in A. Quadrant values indicate percentages of gated targets. In all experiments, CD4^+^ T cell lines were used as targets. CD8^+^ T cells that had been stimulated with SIV-infected targets for 6 days were used as effectors. GrB target cell activity and ICE were calculated after 1 hour of incubation of effectors and plated at an E∶T ratio of 25∶1.

To measure CD8^+^ T-cell cytotoxic capacity, we adapted use of these CD4^+^ T-cell lines to our assay previously used in humans [12]–[14]. Briefly, autologous macaque CD4^+^ T-cell lines were infected with SIV and mixed with PBMC for 6 days to stimulate effectors. In prior work in humans, we observed that this stimulation provided optimal loading with cytolytic molecules and cytotoxicity. CD8^+^ T cells were then negatively isolated and mixed with a second aliquot of surface labeled infected or uninfected autologous CD4^+^ T-cell lines. This mixing is performed in the presence of a cell permeable GrB substrate that fluoresces upon delivery of active GrB to target cells. After 1 hour, GrB delivery was measured by flow cytometry (Figure 1). The remaining cells were fixed, permeablized, and stained for CD4 and intracellular SIV p27 antigen. Cytotoxicity was measured as the fraction of targets to which active GrB was delivered, and the fraction of HIV infected targets that were eliminated (ICE) in a 1-hour period.

Personnel who were blinded to the clinical history and level of *in vivo* viral control of each animal evaluated samples from 23 rhesus macaques with variable levels of control over SIV replication and rates of disease progression. To provide a balanced comparison and control for the effects of MHC, the progressor group was enriched for protective MHC class I alleles. Thus, the frequency of the Mamu class I alleles A*01, B*08 or B*17 did not differ between LTNP/EC and progressors (72.7% (8/11) versus 54.6% (6/11), respectively, p>0.5).

In 22 of 23 animals, significant CD8^+^ T-cell cytotoxic responses were detectable by flow cytometry (Figure 1). In a subset of 7 macaques in which 2 measurements were performed under identical conditions on samples obtained from the same time points, reproducibility of the GrB and ICE results was confirmed to be high since the median paired differences between the first and second measurements were not significantly different from zero (GrB target cell activity: 2.3% [−10.8–13.4%], p>0.5; ICE: −1.0% [−16.8–8.9%], p = 0.31), which is consistent with our observations in humans [12]–[14].

Macaques appeared to fall within 4 subgroups based on the level of SIV-specific CD8^+^ T-cell cytotoxicity as measured by the ICE assay, which has a broad dynamic range. Presumptive assignment of disease status was, therefore, arbitrarily based on this blinded analysis and the previously demonstrated strong association between ICE values and control over HIV replication in humans [12]–[14]. “High responder” subjects with ICE values of ≥50% were provisionally categorized as LTNP/EC. “Low responders” with ICE values of ≤30% were provisionally categorized as progressors. Subjects with ICE between 30% and 50% were considered to have intermediate control of SIV replication, consistent with a slow progressor phenotype. A subject was considered SIV-negative if the CD8^+^ T cells did not exhibit SIV-specific killing and secrete IFN-γ after incubation with SIV-infected targets. As had been observed in HIV infection, killing was remarkably rapid with the CD8^+^ T cells of the “high responders” eliminating most SIV-infected targets within only 1 hour of co-incubation (Figure 1, top row). In contrast, CD8^+^ T cells of the “low responders” eliminated fewer SIV-infected targets within the same time period (Figure 1, bottom row).

The criteria used to classify the individuals based on disease outcome were the following: LTNP/EC were animals with a clinically healthy course, negative history for opportunistic diseases, stable CD4 counts, set point plasma SIV RNA levels <10,000 copies/ml, and no ongoing antiretroviral therapy. Progressor macaques had a history of opportunistic diseases, declining CD4 counts and/or set point plasma SIV RNA levels of >75,000 copies/ml. Slow progressor criteria included nonprogressive disease during at least the first year of SIV infection and plasma SIV RNA levels >10,000 copies/ml. Currently there is no consensus regarding case definitions for LTNP/EC and progressor macaques, nor thresholds identifying which animals will develop immunodeficiency and which ones will not. Therefore, the aforementioned definitions, although somewhat arbitrary, were selected in an attempt to classify animals with clearly different disease outcomes after SIV infection. Where pooled PBMC from more than one time point were required to obtain sufficient cell numbers, CD4 counts and plasma viral loads were reported as the mean of 3 determinations within a 6-month period that included the time points of the samples used in the experiments. Upon unblinding, the disease outcome of the 23 rhesus macaques was accurately predicted in 19 (83%) of the cases (Table 1). The weighted kappa coefficient, which adjusts for agreement by chance, indicated good agreement between our prediction and the disease status, with a value of 0.75 (95% confidence interval (CI): 0.52–0.99, p<0.001) [27]. As another means to assess reproducibility of ICE measurements, agreement in predictions of disease status based on the 2 sets of ICE measurements that were performed under identical conditions in the small subset of 7 animals was also determined by the weighted kappa statistic. Predicted disease status based on the 2 measurements did not change in 6 of the 7 animals. In the last case, macaque C59Z was predicted to be a slow progressor based on an ICE value of 32% and a progressor based on a second result of 23.1%. Therefore, agreement in predicted disease status between the 2 sets of ICE measurements was high with a weighted kappa of 0.84 (95% CI: 0.55–1.0, p = 0.02), which further supports that ICE measurements are highly reproducible.

**Table 1:** Characteristics of macaques and prediction of SIV control.

Subject	SIV strain	ICE (%)	Infection (years)	CD4^+^ T cells/mL	SIV RNA Levels (copies/mL)	Prediction	Disease status
D545	SIVmac239	63.7	7.5	874	<10	LTNP/EC	LTNP/EC
A94	E660, SIVmac 239	91.7	8.7	417	150	LTNP/EC	LTNP/EC
C20	SIVmac239	67.2	6.2	1120	56	LTNP/EC	LTNP/EC
01027	SIVmac239	74.2	0.7	1327	4600	LTNP/EC	LTNP/EC
00032*	SIVmac239	76.9	1.0	1796	320	LTNP/EC	LTNP/EC
AU10*	SIVmac239	67.3	0.5	1201	190	LTNP/EC	LTNP/EC
DBCE*	E660	76.6	0.8	2475	3400	LTNP/EC	LTNP/EC
98016	SIVmac239	77.3	6.7	N/A	5400	LTNP/EC	LTNP/EC
C114*	SIVmac239	58	9.2	709	19000	LTNP/EC	SP
A98	E660, SIVmac239	41	6.5	966	260	SP	LTNP/EC
C59Z*	SIVmac2339	32	7.5	918	13000	SP	SP
91003*	SIVmac239	43.5	1.1	171	680000	SP	SP
DBGR	E660	40.2	2.2	1237	140000	SP	SP
03016*	SIVmac239	22	2.6	1154	<10	Progressor	LTNP/EC
977Z*	E660	28	8.7	2540	<30	Progressor	LTNP/EC
379	E660	7.6	8.9	320	99000	Progressor	Progressor
AY33*	SIVmac239	0	0.1	1381	5400000	Progressor	Progressor
D391*	E660	23.7	8.4	170	84000	Progressor	Progressor
DBXR*	E660	17.3	0.4	1050	7900000	Progressor	Progressor
AY57*	E660	20.4	1.1	620	17000000	Progressor	Progressor
99019*	SIVmac239	25	0.4	416	2100000	Progressor	Progressor
DBWL*	E660	13.9	0.8	1131	2900000	Progressor	Progressor
DBXL*	N/A	0	N/A	N/A	N/A	SIV negative	SIV negative

ICE, Infected CD4^+^ T cell Elimination (%). LTNP/EC, Long-Term Nonprogressor/Elite Controllers. SP, Slow Progressor. N/A, Not Applicable. PBMC from more than one time point were used in the animals marked with an asterisk.

Given the small number of slow progressors, they were included in the progressor group in subsequent analyses. LTNP/EC tended to be infected with SIV for longer durations than progressors (medians, 6.2 versus 1.1 years, respectively), although this did not achieve statistical significance (p>0.5). Even though median CD4 counts were not significantly different between LTNP/EC and progressors (1,178 versus 709 cells/mL, respectively, p = 0.06), median SIV RNA levels, as expected, were significantly lower in LTNP/EC compared with progressors (190 versus 680,000 copies/mL, respectively, p<0.001). These viral RNA levels were comparable to those reported in other groups of LTNP/EC and progressor macaques [16], [22]. In addition, the differences in SIV RNA levels between LTNP/EC and progressors were substantial since the most viremic LTNP/EC macaque in our study had SIV RNA levels that were still 15-fold lower than those of the least viremic progressor. These findings support that the parameters used in our selected case definitions were consistent with those reported previously and accurately categorized macaques based on their disease status.

High backgrounds of GrB activity and ICE were observed in some of the animals during the first experiments, which were attributed to macaque CD8^+^ T cells reacting against xeno-antigens from residual human feeder cells that were used to maintain the CD4^+^ T-cell lines. This background decreased significantly after depleting the human feeders before the 6-day stimulation of rhesus PBMC with the CD4^+^ T-cell lines and the day upon which SIV-specific CD8^+^ T-cell killing activity was measured (data not shown). We then investigated whether the depletion of human feeders from the rhesus macaque-derived CD4^+^ T-cell targets improved the prediction of disease status. In a comparison between the first subgroup of results that had been generated prior to incorporating routine depletions and the second subgroup in which this optimization step had been performed, the percent correctly identified was not significantly different (70% (7/10) versus 92.3% (12/13), respectively; p = 0.28), which might relate to the relatively small sample sizes of each subgroup. However, when the 4 macaques from the first subgroup with moderately high backgrounds (A94, 977Z, A98, C114) were excluded, the accuracy of our prediction for the remaining 19 macaques increased to an even higher level of weighted kappa agreement (weighted kappa, 0.90; 95% CI: 0.72–1.00; p<0.001). Taken together, these data demonstrate that SIV-specific CD8^+^ T-cell cytotoxic capacity can be used to accurately predict the disease status and the level of SIV control in rhesus macaques.

### SIV-specific CD8^+^ T-cell cytotoxicity mediated by the granule exocytosis pathway is associated with immunological control in SIV infection and correlates inversely with plasma SIV loads

**Figure 2:**
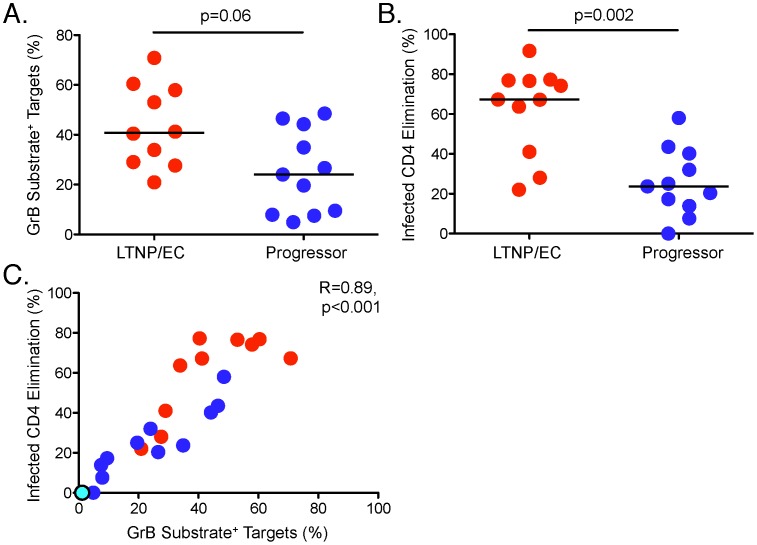
SIV-specific CD8^+^ T cells from LTNP/EC mediate greater lysis of SIV-infected CD4^+^ T-cell targets compared with progressors. GrB target cell activity (**A**) and infected CD4 elimination (ICE) (**B**) are shown for LTNP/EC (n = 10, GrB target cell activity; n = 11, ICE) and progressors (n = 11). Horizontal bars represent the median values. **C.** Correlation between ICE and GrB target cell activity (n = 22) was determined by the Spearman rank method. Red, blue and cyan dots represent LTNP/EC, progressors and one SIV-uninfected animal, respectively.

In comparisons between LTNP/EC and progressors, differences in the median delivery of active GrB to SIV-infected targets did not achieve statistical significance (40.8% [20.9–70.8%] versus 24% [4.9–48.5%], respectively, p = 0.06, Figure 2A). In contrast, the median ICE of LTNP/EC was significantly greater than that of progressors (67.3% [22.0–91.7%] versus 23.7% [0.0–58%], respectively, p = 0.002, Figure 2B). Nearly all of the target cells killed in the 1-hour assay based on light scatter characteristics were GrB substrate positive (data not shown), as observed in humans [12]–[14]. It is noteworthy that in 2 of the 3 LTNP/EC, low cytotoxic responses were attributable to higher backgrounds in samples measured prior to adopting routine depletion of human feeder cells. In addition, one progressor with moderately elevated background in the GrB assay also had a high ICE response in the range of LTNP/EC. Of note, this progressor had been an LTNP/EC for 9 years, but was exhibiting loss of control with higher SIV RNA levels at the time samples were obtained for use in these experiments.

In order to determine whether the differences in ICE between LTNP/EC and progressors were independent of the infecting SIV strain, we compared ICE between SIVmac239-infected (n = 12) and SIVmacE660-infected (n = 8) animals. The median ICE was not significantly different between these groups (60.9% [0–77.3%] versus 22.1% [7.6–76.6%], respectively, p = 0.12; data not shown). Furthermore, the median percent of SIV-infected targets in LTNP/EC and progressors was high and not statistically different (61.4% [45.4–92.6%] versus 44.6% [30.0–83.1%], respectively, p = 0.07; data not shown), indicating that differences in ICE could not be attributed to variability in target cell numbers or their susceptibility to infection.

**Figure 3:**
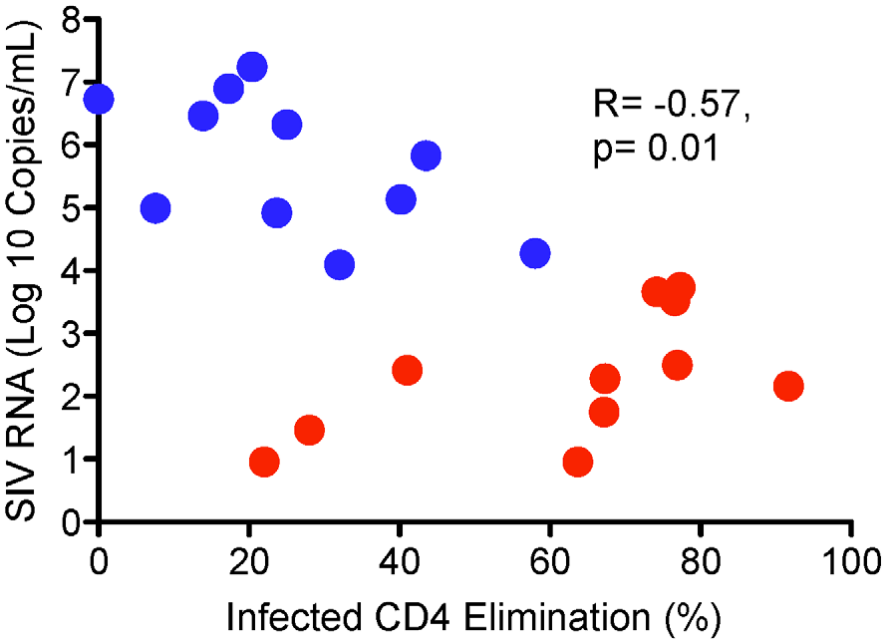
Infected CD4 Elimination (ICE) inversely correlates with plasma SIV RNA levels in SIV-infected rhesus macaques. Red dots represent LTNP/EC (n = 11). Blue dots represent progressors (n = 11). Statistical analysis was performed by the Spearman rank method.

We found a strong correlation between delivery of active GrB to SIV-infected targets from stimulated CD8^+^ T cells and ICE (r = 0.89, p<0.001; Figure 2C) consistent with prior work in humans [12]–[14]. This correlation remained strong when the SIV-negative animal was excluded from the analysis (r = 0.87, p<0.001). In addition, plasma SIV RNA levels were inversely correlated with ICE (r = −0.57, p = 0.01; Figure 3). In a post-hoc analysis, we excluded the animals whose target cells did not undergo depletion of human feeders and at the same time had moderately high GrB background. These macaques (A94, 977Z, A98, C114) represented 3 of the 4 animals that were misclassified. The median differences in the cytotoxic responses between LTNP/EC and progressors increased: GrB target cell activity, 47.1% [20.9–70.8%] versus 21.8% [4.9%–46.5%] (p = 0.02) and ICE, 70.8% [22.0–77.3%] versus 22.1% [0.0–43.5%], respectively (p = 0.002). Additionally, the correlation coefficient comparing SIV RNA levels with ICE values increased (r = −0.63, p = 0.01; data not shown).

Overall, these findings indicate that SIV-specific CD8^+^ T cells optimally kill SIV-infected targets by the granule exocytosis pathway following loading of lytic granule contents, as has been shown in human HIV infection [12]–[14]. Most importantly, they also support the idea that LTNP/EC and progressor rhesus macaques can be distinguished by their SIV-specific CD8^+^ T-cell cytotoxic capacity, suggesting that this parameter is a potential candidate for a correlate of protective immunity in the rhesus macaque SIV infection model.

### SIV-specific CD8^+^ T cells of LTNP/EC mediate greater per-cell killing of SIV-infected targets than those of progressors

**Figure 4:**
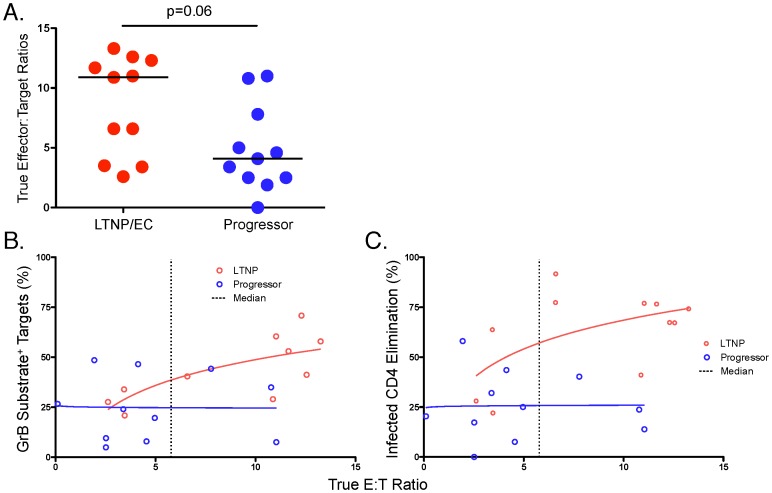
SIV-specific CD8^+^ T cells of LTNP/EC mediate greater per-cell killing of SIV-infected targets than those of progressors, which is not simply due to higher true E∶T ratios. **A.** The true effector to target (E∶T) ratios, determined by measurements of IFN-γ-secreting CD8^+^ T-cell effectors and p27-expressing CD4^+^ T-cell targets, respectively, as described in the Methods and shown in the Figure S1 and Table S1, were compared between LTNP/EC (n = 11) and progressors (n = 11). Horizontal bars represent the median values. **B, C.** GrB target cell activity (**B**) or ICE (**C**) responses plotted against the true E∶T ratios are shown for LTNP/EC (n = 10, GrB target cell activity; n = 11, ICE) and progressors (n = 11). GrB target cell activity is shown after subtraction of background. The response curves were analyzed by regressing ICE and GrB on log true E∶T ratios using analysis of covariance. The standard two-tailed t test from regression analysis was used to compare estimated GrB target cell activity and ICE of LTNP/EC with that of progressors at the 5.8 E∶T ratio, the median of the combined E∶T ranges of both groups.

In order to determine whether the diminished cytotoxic responses of progressors relative to LTNP/EC were merely due to lower CD8^+^ T-cell numbers following 6 days of stimulation or also reflected reduced per-cell cytotoxic capacity, we analyzed the SIV-specific responses over the range of true effector-to-target (E∶T) ratios based on the percentage of IFN-γ-secreting effectors and p27-expressing targets (Figure S1, Table S1 and Figure 4) [12]–[14]. Although the true median E∶T ratios determined by these measurements were not significantly different between LTNP/EC and progressor macaques (10.9% [2.6–13.3%] versus 4.1% [0.0–11.0%], respectively, p = 0.06; Figure 4A), a trend towards higher E∶T ratios was observed among LTNP/EC, which is likely due to their known increased CD8^+^ T-cell proliferative capacity. To further assess whether differences in per-cell cytotoxic capacity also existed between LTNP/EC and progressors, killing curves representing the trends for each group were generated by plotting the cytotoxic responses against these true E∶T ratios. Differences between the killing curves were then quantified by regression analysis and analysis of covariance at the median log E∶T ratio. Non-overlapping curves would support the existence of differences in per-cell cytotoxic capacity (in addition to differences in cell numbers), whereas overlapping curves would support the low cytotoxicity of progressor CD8^+^ T cells was primarily due to fewer cell numbers following expansion since per-cell cytotoxic capacity was similar to that of LTNP/EC. The capacity of CD8^+^ T cells of LTNP/EC to deliver active GrB to infected targets on a per-cell basis was not significantly different from that of progressors at the median E∶T ratio of 5.8 (39% versus 25%, p = 0.14; Figure 4B). However, LTNP/EC-derived CD8^+^ T cells displayed significantly higher ICE on a per-cell basis than did those of progressors at the median E∶T ratio of 5.8 (57% versus 26%, p = 0.004; Figure 4C). Notably, the cytotoxic responses of progressors measured by either parameter plateaued at low E∶T ratios and did not increase significantly even at high E∶T ratios, suggestive of limited per-cell cytotoxic capacity. This is in marked contrast to the responses of LTNP/EC (Figures 4B and C). In summary, these results indicate that CD8^+^ T cells of LTNP/EC possess greater SIV-specific cytotoxic capacity on a per-cell basis than those of progressors and that changes in both the numbers of virus-specific CD8^+^ T cells and in their per-cell cytotoxic capacity following re-stimulation with SIV-infected targets are required for maximal cytotoxicity, as had been observed in human HIV infection [12]–[14].

### SIV-specific CD8^+^ T cells can be expanded after treatment with phorbol ester and calcium ionophore

**Figure 5:**
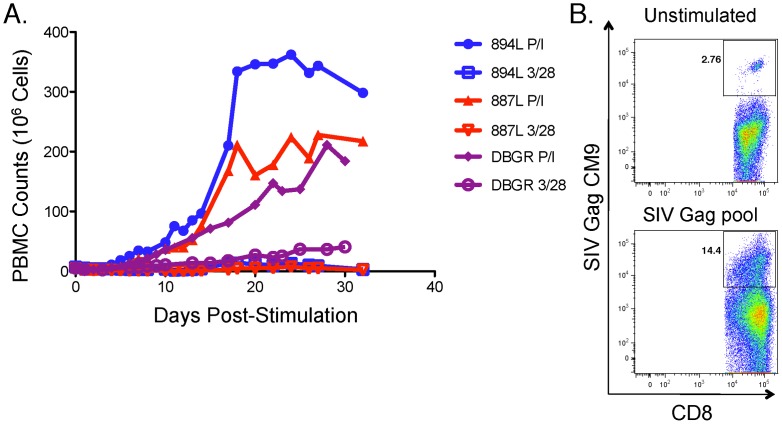
PBMC from progressors can be expanded *in vitro* after treatment with phorbol esther and calcium ionophore. **A.** Three Mamu A*01^+^ rhesus macaques (894L, 887L, DBGR) were stimulated for 6 hours with phorbol-12-myristate-13-acetate (PMA, 400 ng/ml) and ionomycin (Io, 2 µM) on day 0, washed, re-suspended in medium containing 40 IU/mL of IL-2 and cultured for at least 30 days. Top medium was replaced with fresh IL-2-containing medium and counted every 24–48 hours. The controls were not treated with PMA/Io on day 0 but were stimulated with anti-CD3 and anti-CD28 monoclonal antibodies during the first 6 days. **B.** PBMC from another Mamu A*01^+^ rhesus macaque were stimulated with the same concentrations of PMA/Io for 6 hours on day 0 and propagated in culture for an additional 18 days as described above. A pool containing 15-mers spanning the entire SIV Gag sequence was used to stimulate half of the cells on day 18. PBMC were stained on day 24 with the Mamu A01-restricted CTPYDINQM (CM9) tetramer to measure SIV-specific expansion.

It remained unclear whether the cytotoxic capacity of SIV-specific CD8^+^ T-cells from progressors was permanently lost through exhaustion, deletion, or replicative senescence [28]–[31]. In prior work in humans, we did not observe increases in cytotoxic capacity of CD8^+^ T cells of progressors following stimulation with IL-2, IL-15, or stimulation through CD28 and the T-cell receptor [12]. However, we did observe that bypassing TCR stimulation by treatment with phorbol 12-myristate 13-acetate and ionomycin (PMA/Io) followed by a period of rest can restore the proliferative and cytotoxic defects of HIV-specific CD8^+^ T cells *in vitro*. [12]. Therefore, we explored whether PBMC of progressor rhesus macaques could similarly be expanded by treatment with PMA/Io *in vitro*. PBMC from 3 additional Mamu A01^+^ progressors (894L, 887L and DBGR) were stimulated with either 400 ng/mL of PMA and 2 µM of ionomycin or anti-CD3 and anti-CD28 monoclonal antibodies (Figure 5A). In preliminary experiments, it had been determined that these concentrations of PMA/Io induced maximal expansion following 6-hour stimulation and that medium containing exogenous IL-2 was required to support prolonged *in vitro* propagation of PBMC (data not shown). Strikingly, the median peak expansion in the cultures treated with PMA/Io was 224-fold [range: 106–241], which occurred during the third week (Figure 5A). In contrast, the median expansion was only 3-fold [range: 3–12] in the anti-CD3/CD28-stimulated controls.

In addition, PBMC from another macaque were expanded after a 6-hour stimulation with PMA/Io. A pool containing 15-mers spanning the entire SIV Gag sequence was added to half of the cells on day 18. On day 24, PBMC were stained with the Mamu A01-restricted SIV Gag CTPYDINQM tetramer. Gag-stimulated cells demonstrated a 5-fold expansion over the unstimulated control cells (Figure 5B). After demonstrating antigen-specific cells could be expanded *in vitro* following PMA/Io treatment, we attempted to examine the cytotoxic capacity of these expanded SIV-specific CD8^+^ T cells of two progressor animals, but the results were inconclusive. We attributed this to the low starting cell numbers and the profound baseline immunodeficiency of these particular macaques. Unfortunately, sufficient cell numbers from other progressor macaques who were at a less advanced stage of disease were not available. In summary, the proliferative defect of SIV-specific CD8^+^ T cells observed in progressors is reversible and can be restored with potent stimulation *in vitro* followed by a period of rest, as observed previously in humans [12].

## Discussion

In this study, the cytotoxic capacity of SIV-specific CD8^+^ T cells in response to SIV-infected CD4^+^ T-cell targets was measured in blood samples from rhesus macaques with different degrees of control over *in vivo* SIV replication. Consistent with some prior work in humans [12]–[14], the SIV-specific CD8^+^ T cells of LTNP/EC were found to have increased cytotoxic capacity compared with those of progressors. Further, the ability of SIV-specific CD8^+^ T cells of LTNP/EC to eliminate SIV-infected CD4^+^ T cells was mediated by delivery of active GrB to target cells. These results provide a CD8^+^ T-cell function and potential mechanism that distinguish LTNP/EC from progressors and appear to represent a correlate of immune control in chronic SIV infection.

Although a clear correlate of immune control in chronic SIV infection has not been demonstrated in prior work [21]–[23], there are a number of important differences with the present study. In a previous study, a viral suppression assay was developed to investigate the responses of elite controller and progressor macaques, but it did not accurately predict immune control [23]. However, there are two important technical differences that may explain the discrepant results of the two studies. First, the prior study used sorted tetramer-positive effector cells in contrast to polyclonal CD8^+^ T cells used in the present study. In prior work, we did not observe robust differences in proliferation or cytotoxic capacities of HIV-specific CD8^+^ T-cells between patient groups when we used single peptides or peptide pools covering a single gene product. The largest differences were found when we used HIV-infected cells that sample the cytotoxic capacities of a broader range of specificities covering the HIV proteome [12]. Second, in the prior study effectors were stimulated for 2 days versus 6 days in the present study [23]. In our prior work in humans, we did observe small but statistically significant differences in cytotoxic capacities of CD8^+^ T cells between progressors and LTNP/EC in the absence of effector cell restimulation. However, differences were much more robust after 6 days of stimulation, with limited or no overlap between both groups. This restimulation allows CD8^+^ T cells of LTNP/EC, which likely have limited exposure to antigen *in vivo*, to fully load lytic granules [12]–[14]. In one recent study in vaccinated macaques, greater pre-challenge SIV-specific CD8^+^ T cell suppressive capacity was observed in animals with modest virologic control compared to those with poor control, post-SIVsmE660 challenge [32]. Suppressive capacity is sampled over 5–14 days in these assays, allowing ample time for lytic granule loading under conditions where antigen is limited, such as it occurs in LTNP/EC and vaccination with non-persisting vectors.

The results of the present study provide some additional insight regarding the mechanisms of immunologic control over SIV in rhesus macaques. Our observations are consistent with previous evidence indicating that CD8^+^ T cells play a fundamental role in the restriction of SIV infection *in vivo* [15]–[20]. Both measures of CD8^+^ T-cell-mediated killing, ICE and GrB cell activity, strongly correlated with each other in this study. Furthermore, based on light scatter characteristics, we did not observe a significant population of killed SIV-infected target cells that lacked active GrB (data not shown). Diminished killing by progressors' CD8^+^ T cells was not due to death of effector cells because 6 hours later they were still viable and produced IFN-γ. These findings and the demonstrated association between immunologic control of SIV replication and increased per-cell SIV-specific CD8^+^ T cell cytotoxic capacity, similarly observed in chronic HIV infection in humans, suggests that the granule exocytosis pathway is likely an important mechanism for controlling not only HIV, but other lentiviruses including SIV [12]–[14].

Although our study suggests that CD8^+^ T cell cytotoxic capacity is associated with disease outcome in SIV-infected animals and potentially in vaccinees [32], some technical challenges remain. Performing these assays required generation of autologous hTERT-transduced CD4^+^ T cell lines that were maintained using human feeder cells. These cell lines were then used to stimulate rhesus macaque PBMC for 6 days. High backgrounds observed in initial experiments likely reflected xeno-reactivity of macaque CD8^+^ T cells against residual human antigens from irradiated human feeder cells that were used to support the hTERT-transduced macaque CD4^+^ T cell lines. This may explain why a few macaques were misclassified. Four animals (A94, 977Z, A98 and C114), including 3 that were misclassified, had unusually high background GrB target cell activity because the experiments were conducted prior to instituting routine depletion of human feeders from the CD4^+^ T-cell lines, before the 6-day PBMC stimulation and the day upon which SIV-specific CD8^+^ T-cell killing activity was measured. Excluding these 4 macaques did improve the accuracy of our prediction to an even higher level of weighted kappa agreement (from 0.75 to 0.90). Of note, even though animal C114 had a relatively high ICE response, it was defined as a progressor based on clinical data. However, this animal had an unusual natural history, having remained healthy for 9 years following SIV infection until finally succumbing to disease at the time samples available for evaluation of cytotoxic capacity were obtained. The relatively high ICE response seen may be related to the fact that animal C114 controlled infection for 9 years and was just starting to experience progressive disease. Therefore, its SIV-specific cytotoxic capacity was concordant with the disease outcome. Overall, differences in cytotoxic capacity between groups were less robust in the present study compared to prior work in humans. This is possibly related to the use of cell lines, allogeneic human feeders, and the additional manipulation required compared to the assay in humans. Further refinements, such as conducting this assay on a smaller scale, may obviate the need for hTERT-transduced targets and human feeders, and allow for measurement of cytotoxic capacity with less variability.

In this study, there were 3 LTNP/EC macaques (A98, 03016, 977Z) that had low CD8^+^ T-cell cytotoxic capacity. Although this was attributed to high background in the GrB assay prior to depletion of human feeders for A98 and 977Z, this explanation did not apply in the case of 03016. Similarly, we and other groups have infrequently observed human LTNP/EC with low CD8^+^ T-cell cytotoxic capacity [33], [34]. Therefore, even though most studies to date indicate that the large majority of LTNP/EC control lentiviral replication by CD8^+^ T-cell mediated cytotoxicity, it remains possible that a small portion of these individuals might control lentiviral replication by alternative mechanisms.

Our results document that SIV-specific CD8^+^ T cells can be stimulated through cell cycle and expanded. In prior work in humans, diminished proliferative and cytotoxic capacities of HIV-specific CD8^+^ T-cells of progressors could be restored by *in vitro* treatment with PMA/Io followed by a period of rest. However, similar expansion was not observed after stimulation through the T-cell receptor (TCR) using anti-CD3 and anti-CD28 monoclonal antibodies [12]. In the present study, we observed similar characteristics of SIV-specific CD8^+^ T-cells of progressor rhesus macaques. Although PMA/Io bypasses the TCR by causing intracellular calcium flux, its mechanism of action in this context remains incompletely understood. PMA/Io has been identified as one of the stimuli reported to overcome the anergic state [31], [35]–[37]. Its function in restimulating T cells may be to bypass or override TCR stimulation. Following PMA/Io stimulation and a period of rest, progressor CD8^+^ T cells respond to HIV infected CD4^+^ T cells by proliferating and killing without additional cytokines or other stimuli [12]. Reversal of defective cytotoxicity of progressor macaque SIV-specific CD8^+^ T-cells was not demonstrated in the present study; however, this line of investigation remains of interest considering its significant implications for the design of effective immunotherapies.

It should be noted that an association between ICE and disease status does not establish causality. Although causality has not been directly demonstrated here or in humans, prior work strongly suggests that diminished cytotoxic capacity is not just an effect of high viral load in progressors. Reducing viral load by antiretroviral therapy does not restore cytotoxic capacity to HIV-specific CD8^+^ T-cells [12], [13]. Direct proof of a causal role of cytotoxic capacity might require passive transfer of functional SIV-specific CD8^+^ T cells to progressor macaques. Prior attempts to reduce the viral load in non-human primates by the passive transfer of autologous SIV-specific CD8^+^ T-cell clones have been unsuccessful [38], [39]. However, it remains possible that a polyclonal population of autologous virus-specific CD8^+^ T cells with documented cytotoxic capacity, as occurs in LTNP/EC *in vivo*, could potentially reduce the viral load.

Most importantly, our results have some implications for HIV vaccines. Although a humoral immune response will likely be a critical component of an efficacious HIV vaccine in humans, a cellular immune response will likely be necessary to control viremia in breakthrough infection of vaccinees [14]. Therefore, a further understanding of natural control of HIV and SIV may provide important information regarding the targets and qualities of a successful cellular immune response capable of controlling the virus that may be stimulated by a vaccine. Given the correlation between cytotoxic capacity and virologic control of SIV infection demonstrated here, measuring cytotoxic capacity in response to different SIV viruses, following vaccination, could be very useful to screen candidate vaccines. The results of the present study provide an immunological function that correlates with control over SIV replication that will help understand how tests in development could be used as correlates of immunologic control in immunized macaques.

## Materials and Methods

### Ethics statement

Animals were housed and cared in accordance with standards of the Association for Assessment and Accreditation of Laboratory Animal Care (AAALAC) in AAALAC accredited facilities, and all animal procedures were performed according to protocols approved by the Institutional Animal Care and Use Committees of the National Cancer Institute, National Institutes of Health and the Institutional Animal Care and Use Committee of the Graduate School of the University of Wisconsin (Animal Welfare Assurance No. A3368-01).

### Macaques

Twenty-three Indian rhesus macaques (*Macaca mulatta*) were studied, 22 of them infected with Simian Immunodeficiency Virus (SIV). At study initiation, animals were seronegative for simian type D retrovirus and simian T cell lymphotropic/leukemia virus type 1. LTNP/EC criteria included: clinically healthy status, negative history for opportunistic infections or neoplasms, stable CD4 counts, set point plasma SIV RNA levels <10,000 copies/ml, and no ongoing antiretroviral therapy. Progressors were defined as rhesus macaques with set point plasma SIV RNA levels of >75,000 copies/ml, declining CD4 count and/or history of opportunistic diseases. Slow progressor criteria included plasma SIV RNA levels >10,000 copies/ml and nonprogressive disease during at least the first year of SIV infection. In addition, PBMC of 4 Mamu A*01^+^ SIV-infected rhesus macaques with progressive disease were stimulated with PMA/Io.

### Classification of macaques

The personnel who performed the experiments and categorized each subject as an LTNP/EC, slow progressor, progressor or SIV-uninfected macaque (D.M., S.A.M.) were blinded to the disease status and level of *in vivo* control of SIV replication. The provisional assignment was arbitrarily based on the value of ICE activity given the broad dynamic range of this assay parameter and previously demonstrated association with *in vivo* control over HIV infection [12]: a subject was considered to be a “high responder” and LTNP/EC if the CD8^+^ T cells had an ICE of ≥50%. “Low responders” with ICE values of ≤30% were categorized as progressors. Subjects with intermediate ICE values between 30% and 50% were classified as slow progressors. A subject was considered SIV-negative if the CD8^+^ T cells did not exhibit SIV-specific killing and IFN-γ secretion after incubation with SIV-infected targets.

### Generation of CD4^+^ T cell line targets

Autologous CD4^+^ T cell clones were generated from rhesus macaque PBMC as described [25]. Briefly, highly enriched CD4^+^ T cells were isolated from PBMC by negative selection using Miltenyi LD columns and anti-CD8 microbeads followed by positive selection using MS columns and anti-CD4 microbeads (Miltenyi Biotec, Auburn, CA). CD4^+^ T cell clones were obtained after 2-week expansion in limiting dilution cultures containing irradiated human PBMC, IL-2 and anti-CD3 monoclonal antibody (mAb). CD4^+^ T cell clones were transduced with a hTERT/nerve growth factor receptor (NGFR) construct as described [26]. Transduction efficiency was determined by surface staining for NGFR expression using anti-human NGFR-PE (clone C40-1457; BD/PharMingen, San Diego, CA). Transduced cells were further enriched by PE microbead selection (Miltenyi, Biotec Inc.). The resulting hTERT-transduced CD4^+^ T-cell lines were maintained using IL-2 and biweekly anti-CD3 mAb stimulation with irradiated human PBMC and human Epstein Barr virus-transformed B-cells (TM B-LCL) as feeder cells [25], [40].

### SIVmac251-infected autologous CD4^+^ T cell line targets

Autologous TERT-transduced CD4^+^ T-cell lines were polyclonally stimulated with anti-CD3 and anti-CD28 mAbs prior to infection as previously described [11]. CD4^+^ T-cell line lymphoblasts were infected with SIVmac251 bound to ViroMag beads (OZ Biosciences, Marseille, France) for 36 hours and then used in cytotoxicity assays or to stimulate PBMC [12].

### Granzyme B cytotoxicity assay

Human feeder cells were depleted from the CD4^+^ T cell lines by labeling them with anti-CD45 microbeads (Miltenyi Biotec) that react with human but not macaque leukocytes. This was performed before the 6-day stimulation of PBMC with SIV-infected CD4^+^ T-cell lines and the day upon which SIV-specific CD8^+^ T-cell killing activity was measured. PBMC effectors co-incubated with SIV-infected targets for 6 days underwent negative selection of CD8^+^ T cells (non-human primate CD8^+^ T cell Isolation Kit II, Miltenyi Biotec). CD8^+^ T cells were not isolated from unstimulated PBMC since their cytotoxic function has not been observed to distinguish LTNP/EC from progressors [12], [23]. Targets were labeled with a LIVE/DEAD Fixable Violet Stain Kit (Molecular Probes/Invitrogen Detection Technologies, Eugene, OR). CD8^+^ T cells were combined with targets at an E∶T ratio of 25∶1 for 1 hour at 37°C in the presence of the granzyme (Gr) B substrate. This E∶T ratio was chosen based on prior observations in humans [12]–[14] and in preliminary experiments with macaque cells demonstrating an optimal signal to noise ratio at this E∶T for LTNP/EC and progressors (data not shown). The GranToxiLux killing assay was conducted per the manufacturer's protocol (OncoImmunin, Inc.) with minor revisions [12], [14]. Following analysis of GrB activity by flow cytometry, cells were treated with Cytofix/Cytoperm (BD Biosciences, San Jose, CA) prior to staining to confirm infection and to measure elimination of p27-expressing cells. Infected CD4 elimination (ICE) was calculated as follows: [(%p27 expression of infected targets) minus (%p27 expression of infected targets mixed with D#6 effector cells) divided by (%p27 expression of infected targets)]×100. To assess per-cell cytotoxic capacity, cytotoxic responses were plotted against the true E∶T ratios, as described previously [12]–[14]. True E∶T ratios were based on parallel measurements of true effector cell numbers determined by the frequencies of IFN-γ^+^ CD8^+^ T cells, and on true target numbers determined by the total percentages of p27^+^ CD3^+^ cells.

### CD8^+^ T cell stimulation assays for intracellular protein detection

In experiments using CD4^+^ T-cell line targets to measure the total frequency of virus-specific CD8^+^ T cells, CD8^+^ T cells were co-incubated with uninfected or SIVmac251-infected autologous CD4^+^ T-cell line targets at an E∶T ratio of 1∶1. This E∶T ratio differs from the 25∶1 E∶T ratio used in replicates to measure cytotoxicity because the goal was to maximize the stimulation conditions to a point of saturation in order to accurately enumerate all of the SIV-specific effectors present. An E∶T ratio of 1∶1 was found to be optimal in preliminary experiments [12], [13]. At 2 hours, brefeldin-A (10 mg/ml; Sigma Aldrich, St. Louis, MO) was added to inhibit cytokine secretion. At 6 hours, the cells were washed and stained with surface antibodies prior to fixation, permeabilization and intracellular staining as previously described [12].

### Expansion of PBMC with phorbol ester and calcium ionophore

PBMC were resuspended in 10% FBS medium to a concentration of 4×10^6^ cells/ml and stimulated with phorbol-12-myristate-13-acetate (PMA, 400 ng/ml; Calbiochen, Darmstadt, Germany) and ionomycin (Io, 2 µM; Sigma Aldrich, St. Louis, MO, USA) or anti-CD3 (clone SP34-2; BD Biosciences) and anti-CD28 (1 µg/ml; BD Biosciences) monoclonal antibodies at 37°C. At 6 hours, cells were incubated with benzonase nuclease (125 U/ml; EMD Chemicals, San Diego, CA, USA) at 37°C, washed, resuspended in 10% FBS medium with 40 IU/ml of IL-2 either without (in the case of PMA/Io stimulated) or with anti-CD3/CD28 antibodies (controls). Cells were then incubated in T-25 flasks at 37°C for 6 days. Fresh IL-2 medium was replaced at least every other day in both conditions for up to 30 days. In a different experiment, pooled SIV Gag peptides were added on day 18 and incubated for an additional 6 days prior to tetramer staining to measure SIV-specific expansion.

### Flow cytometry

Multiparameter flow cytometry was performed according to standard protocols. Surface and/or intracellular staining was performed using the following antibodies from BD Biosciences unless otherwise specified: fluorescein isothiocyanate (FITC) or PE Cy7-conjugated anti-CD45 (to gate out human cells); PE-conjugated anti-CD8, and anti-CD4; peridinine chlorophyll protein (PerCP)-conjugated anti-CD3; allophycocyanin (APC)-conjugated anti-IFN-γ (BD Biosciences). Anti-p27 antibodies (Kc57 RDI) were purchased from Beckman Coulter (Fullerton, CA). To measure expansion of SIV-specific CD8^+^ T cells, the Mamu A01-restricted CTPYDINQM (CM9) tetramer was used as described previously [38]. Samples were analyzed on a FACSAria multi-laser cytometer (Becton-Dickinson) with FACSDiva software and 5×10^4^–2×10^6^ CD3^+^ CD8^+^ gated lymphocyte events were collected. In cytotoxicity experiments, 5×10^3^–8×10^3^ target events were collected. Data were analyzed using FlowJo software (TreeStar, San Carlos, CA).

### Statistical analysis

The Wilcoxon two-sample test was used to compare medians and distributions of independent groups. The weighted kappa coefficient was used to measure the level of agreement between the prediction of disease status and the true disease status and between predicted disease status in 7 pairs of ICE measurements. Fisher's exact test was used to compare the frequencies of agreement in independent groups before and after human cell depletion. The Wilcoxon signed rank test was used to determine if the paired differences of repeat measurements of GrB target cell activity and ICE were statistically different from zero. Correlations between viral load, ICE and GrB cell activity were determined by the Spearman rank method. ICE and GrB response curves were analyzed by regressing ICE and GrB on log E∶T ratios using regression and analysis of covariance. The standard two-tailed t test from regression analysis was used to compare estimated ICE and GrB cell activity of LTNP/EC versus progressors at the E∶T ratio of 5.8, the median of the combined E∶T ranges of the whole cohort. The Bonferroni method was used for adjusting all p values for multiple testing, except for the comparison of the frequency of agreement before and after depletion of human feeders that had a single test for a single comparison.

## Supporting Information

Figure S1 **Determination of the percentages of IFN-γ-producing SIV-specific CD8^+^ T cells and the percentages of SIV-infected CD4^+^ T-cell targets.** **A.** IFN-γ expression in the CD8^+^ T cells of two representative LTNP/EC macaques (top two rows) and two representative progressors (bottom two rows) is shown following stimulation for 6 hours, as described in the Methods. Values indicate percentages of gated CD8^+^ T cells. Red values reflect net IFN-γ expression following subtraction of background IFN-γ expression measured in response to uninfected targets (left column) from responses measured against SIVmac251-infected targets (right column). **B.** SIV p27 expression is shown in uninfected (left column) and SIVmac251-infected (right column) CD4^+^ T cell line targets for the same macaques as shown in A. Quadrant values indicate percentages of gated targets. Red values reflect total percentages of SIV p27-expressing targets based on the sum of the upper quadrants of plots depicting infected targets (right column). The red values from **A** and **B** are used to calculate the true E∶T ratio from the plated E∶T ratio for each macaque.
(TIFF)

Table S1 **Determination of true E∶T ratios based upon measurements of IFN-γ-secreting CD8+ T-cell effectors and SIV p27-expressing CD4+ T-cell targets.** Abbreviations are as follows: E, Effectors. T, Targets. LTNP/EC, Long-Term Nonprogressor/Elite Controllers.
(DOCX)
