# Supplementary figures and images for: Cytotoxic Capacity of SIV-Specific CD8+ T Cells against Primary Autologous Targets Correlates with Immune Control in SIV-Infected Rhesus Macaques
Source: PLoS Pathog. 2013 Feb 28;9(2):e1003195. doi: 10.1371/journal.ppat.1003195 (PMC3585127; doi:10.1371/journal.ppat.1003195)

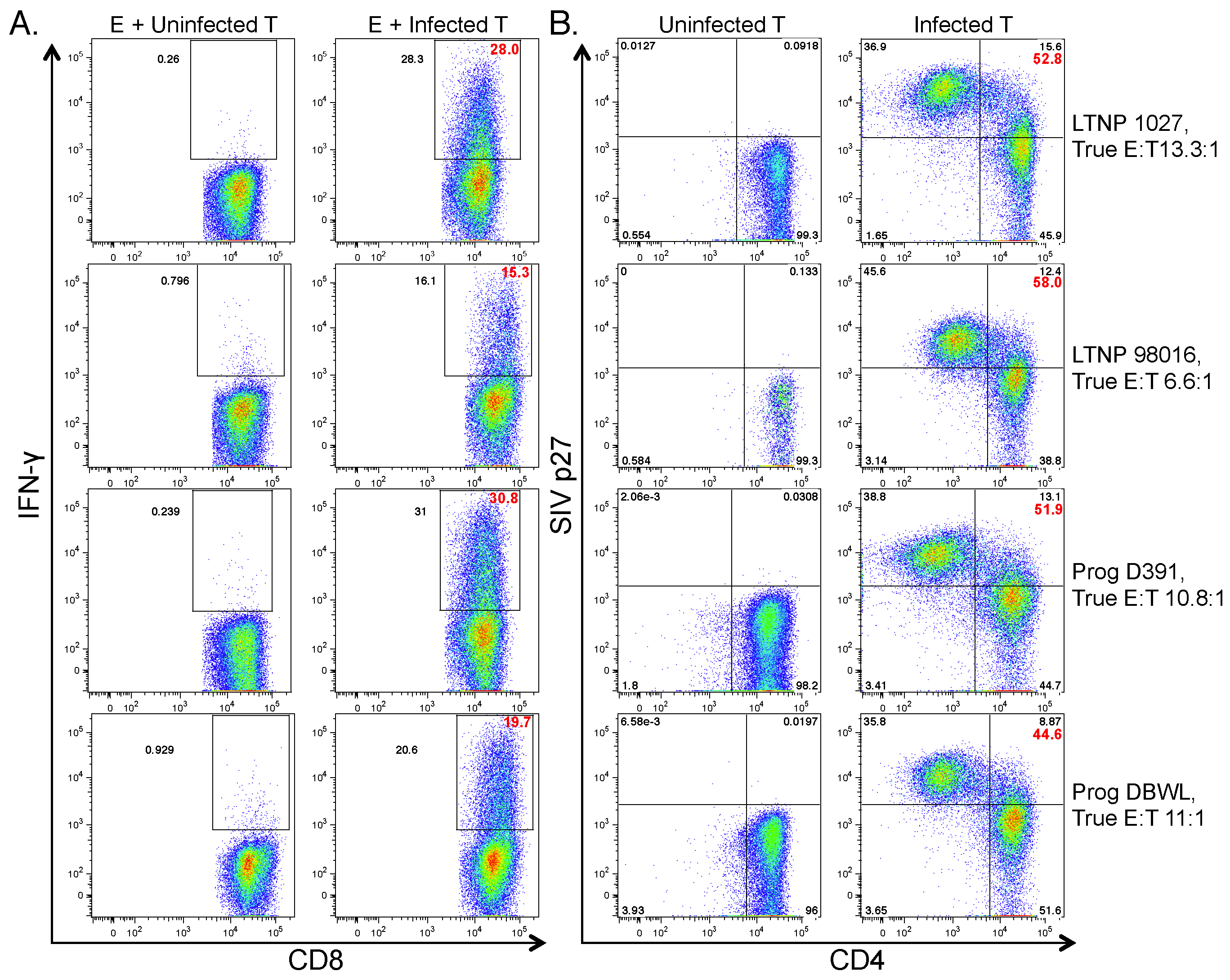

Supplement: Figure S1 — Determination of the percentages of IFN-γ-producing SIV-specific CD8+ T cells and the percentages of SIV-infected CD4+ T-cell targets. A. IFN-γ expression in the CD8+ T cells of two representative LTNP/EC macaques (top two rows) and two representative progressors (bottom two rows) is shown following stimulation for 6 hours, as described in the Methods. Values indicate percentages of gated CD8+ T cells. Red values reflect net IFN-γ expression following subtraction of background IFN-γ expression measured in response to uninfected targets (left column) from responses measured against SIVmac251-infected targets (right column). B. SIV p27 expression is shown in uninfected (left column) and SIVmac251-infected (right column) CD4+ T cell line targets for the same macaques as shown in A. Quadrant values indicate percentages of gated targets. Red values reflect total percentages of SIV p27-expressing targets based on the sum of the upper quadrants of plots depicting infected targets (right column). The red values from A and B are used to calculate the true E∶T ratio from the plated E∶T ratio for each macaque. (TIFF) [file ppat.1003195.s001.tiff]
