# Supplementary material for: Cytotoxic Capacity of SIV-Specific CD8+ T Cells against Primary Autologous Targets Correlates with Immune Control in SIV-Infected Rhesus Macaques
Source: PLoS Pathog. 2013 Feb 28;9(2):e1003195. doi: 10.1371/journal.ppat.1003195 (PMC3585127; doi:10.1371/journal.ppat.1003195)
Supplement: Table S1 — Determination of true E∶T ratios based upon measurements of IFN-γ-secreting CD8+ T-cell effectors and SIV p27-expressing CD4+ T-cell targets. Abbreviations are as follows: E, Effectors. T, Targets. LTNP/EC, Long-Term Nonprogressor/Elite Controllers. (DOCX) [file ppat.1003195.s002.docx]

**Table S1.** Determination of true E:T ratios based upon measurements of IFN-γ-secreting CD8^+^ T-cell effectors and SIV p27-expressing CD4^+^ T-cell targets.

|  | **Subject** | **ICE** | **Plated E:T** | **Plated E** | **IFN-γ^+^CD8^+^ (%)** | **True E** | **Plated T** | **SIVp27^+^ T (%)** | **True T** | **True E:T** |
| --- | --- | --- | --- | --- | --- | --- | --- | --- | --- | --- |
| **LTNP/EC** | D545 | 63.7 | 25:1 | 1250000 | 9.1 | 113750 | 50000 | 66.4 | 33200 | 3.4:1 |
|  | A94 | 91.7 | 25:1 | 1250000 | 21.1 | 263750 | 50000 | 79.9 | 39950 | 6.6:1 |
|  | 977Z | 28 | 25:1 | 1250000 | 5.0 | 63000 | 50000 | 48.1 | 24050 | 2.6:1 |
|  | C20 | 67.2 | 25:1 | 1250000 | 22.8 | 285000 | 50000 | 45.4 | 22700 | 12.6:1 |
|  | 1027 | 74.2 | 25:1 | 1250000 | 28.0 | 350000 | 50000 | 52.8 | 26400 | 13.3:1 |
|  | 32 | 76.9 | 25:1 | 1250000 | 27.1 | 338750 | 50000 | 61.4 | 30700 | 11.0:1 |
|  | AU10 | 67.3 | 25:1 | 1250000 | 45.6 | 569675 | 50000 | 92.6 | 46300 | 12.3:1 |
|  | DBCE | 76.6 | 22.5:1 | 1125000 | 37.5 | 421875 | 50000 | 72.4 | 36200 | 11.7:1 |
|  | 98016 | 77.3 | 25:1 | 1250000 | 15.3 | 191250 | 50000 | 58.0 | 29000 | 6.6:1 |
|  | A98 | 41 | 25:1 | 1250000 | 20.7 | 258750 | 50000 | 47.6 | 23800 | 10.9:1 |
|  | 3016 | 22 | 25:1 | 1250000 | 9.7 | 121250 | 50000 | 70.1 | 35050 | 3.5:1 |
| **Progressors** | C114 | 58 | 25:1 | 1250000 | 2.7 | 33750 | 50000 | 34.8 | 17400 | 1.9:1 |
|  | C59Z | 32 | 22.5:1 | 1125000 | 6.4 | 72000 | 50000 | 42.5 | 21250 | 3.4:1 |
|  | 379 | 7.6 | 16:1 | 625000 | 12.2 | 76250 | 40000 | 41.9 | 16760 | 4.6:1 |
|  | AY33 | 0 | 20:1 | 750000 | 7.1 | 53250 | 37500 | 56.4 | 21150 | 2.5:1 |
|  | D391 | 23.7 | 18:1 | 454200 | 30.8 | 139893 | 25000 | 51.9 | 12975 | 10.8:1 |
|  | DBXR | 17.3 | 20:1 | 1000000 | 6.7 | 67000 | 50000 | 53.1 | 26550 | 2.5:1 |
|  | AY57 | 20.4 | 25:1 | 1250000 | 0.0 | 0 | 50000 | 83.1 | 41550 | 0 |
|  | 99019 | 25 | 24:1 | 1200000 | 8.6 | 103200 | 50000 | 41.6 | 20800 | 5.0:1 |
|  | DBWL | 13.9 | 25:1 | 1250000 | 19.7 | 246250 | 50000 | 44.6 | 22300 | 11.0:1 |
|  | 91003 | 43.5 | 25:1 | 1250000 | 11.2 | 140000 | 50000 | 67.8 | 33900 | 4.1:1 |
|  | DBGR | 40.2 | 25:1 | 1250000 | 9.3 | 116250 | 50000 | 30.0 | 15000 | 7.8:1 |

E, Effectors. T, Targets. LTNP/EC, Long-Term Nonprogressor / Elite Controllers.
